# Intramolecular Conversions of (Aminoferrocenylpenta-1,4-dienyl)-ferrocenylcarbenes: Synthesis of Diferrocenylmono-, bi-, tricycles and Amino(diferrocenyl)hexa-1,3,5-trienes

**DOI:** 10.3390/molecules16075574

**Published:** 2011-06-30

**Authors:** Elena I. Klimova, Luis A. Ortiz-Frade, Miguel A. González-Fuentes, Marcos Flores-Alamo, Leon V. Backinowsky, Marcos Martínez García

**Affiliations:** 1 Facultad de Química, Universidad Nacional Autónoma de México, Cd. Universitaria, Coyoacán, México D.F. 04510, Mexico; Email: mfa24s99@gmail.com (M.F.-A.); 2 Centro de Investigación y Desarrollo Tecnológico en Electroquímica S.C. Parque Tecnológico Querétaro, Pedro de Escobedo, Querétaro 76703, Mexico; Email: laofrade@gmail.com (L.A.O.-F.); 3 N. D. Zelinsky Institute of Organic Chemistry, Russian Academy of Sciences, 47 Leninsky prosp., Moscow 119991, Russia; Email: leon_bakinowsky@mail.ru (L.V.B); 4 Instituto de Química, Universidad Nacional Autónoma de México, Cd. Universitaria, Coyoacán, México D.F. 04510, Mexico; Email: margar@unam.mx (M.M.G.)

**Keywords:** amino(diferrocenyl)cyclopropenylium, cyclopropene ring opening, intramolecular conversion of aminoferrocenylpenta-1,4-dienyl(ferrocenyl)carbenes, 3,4-diferrocenyltoluene, amino(diferrocenyl)bicyclo-[3.1.0]hexanes, amino(ferrocenyl)-ferrocenobicyclo[3.2.1]octenes, amino(diferrocenyl)hexa-1,3,5-trienes, electrochemistry

## Abstract

Synthesis of 3,4-diferrocenyltoluene (**7**), 1-morpholino- and 1-piperidino-2,3-diferrocenylbicyclo[3.1.0]hex-2-enes **8a**, **8b**, 1-morpholino- and 1-piperidino-7-ferrocenyl-3,4-ferrocenobicyclo[3.2.1]oct-6-enes **9a**, **9b**, 2- and 3-amino(diferrocenyl)-hexa-1,3,5-trienes **10a**,**b**, **11a**,**b** by reactions of amino(diferrocenyl)cyclopropenylium tetrafluoro-borates with 1-methylprop-2-enylmagnesium chloride at 80 °C is described. The structures of the compounds obtained were determined by IR, ^1^H- and ^13^C-NMR spectroscopy and mass spectrometry. X-ray diffraction data for 1-piperidino-7-ferrocenyl-3,4-ferroceno-bicyclo[3.2.1]oct-6-ene (**9b**), 2-morpholino- and 2-piperidino-1,3-diferrocenyl-4-methyl-hexa-1,3,5-trienes **10a** and **10b** is presented. The electrochemical behaviour of compounds **7**, **8a**, **10a** and **10b** was investigated by means of cyclic voltammetry and square wave voltammetry. For **7** and **8a** two electrochemical processes (I-II), attributed to the oxidation of the ferrocene moieties were found. On the other hand for compounds **10a** and **10b** a single electron transfer for both ferrocene groups and the electrochemical generation of the monocation and dication species were detected.

## 1. Introduction

Diferrocenylcyclopropenylium cations with heterosubstituents in the small ring have recently been described [1,2,3,4,5,6]. These include 1-ethoxy-, 1-diethylamino-, 1-morpholino-, 1-piperidino-2,3-diferrocenylcyclopropenylium tetrafluoroborates and 2,3-diferrocenyl-1-methylsulfanylcyclo-propenylium iodide. Thus, it was established that the reactions of 1-ethoxy-2,3-diferrocenyl-cyclopropenylium tetrafluoroborate with orghanolithium compounds are regioselective (the nucleophilic attacks of the anionic reagents on cyclopropenylium cation are directed at both C-1 centers of the three-carbon ring) to yield 3,3-dialkyl-1,2-diferrocenylcyclopropenes [1]. We have shown that the reactions of the 2,3-diferrocenyl-1-methylsulfanylcyclopropenylium iodide with RLi and RMgX are nonregioselective to yield diferrocenylcycloropenes in low yields (~10%–l20%), the major products being 2,3 diferrocenyl-1-methylsulfanyl-1,3-dienes and -1,3,5-trienes (~60%–l70%) [4].

**Scheme 1:**
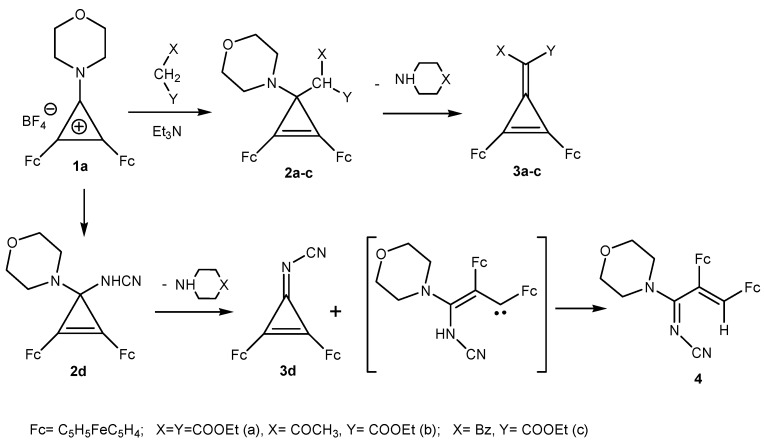
Reaction of 2,3-diferrocenyl-1-morpholinocyclopropenylium tetrafluoroborate **1a** with β-dicarbonyl compounds.

On the whole, salts of the 1-morpholino- or 1-piperidino-2,3-diferrocenylcyclopropenylium series are little studied in both the synthetic and practical aspects. Previously it has been shown that 2,3-diferrocenyl-1-morpholinocyclopropenylium tetrafluoroborate (**1a**) reacted with β-dicarbonyl compounds and sodium cyanamide to afford initially the adducts of the nucleophiles at the small ring, *i.e*., tetrasubstituted diferrocenylcyclopropenes **2a–d** [5,6,7] (Scheme 1).

Elimination of morpholine from cyclopropenes **2a–c** then resulted in 1,2-diferrocenyl-3-(disubstituted)methylidenecyclopropenes **3a–c**. Under the reaction conditions, 3-cyanimino-1,2-diferrocenylcyclopropene (**3d**) and a linear conjugated product **4** formed from compound **2d**. In these reactions, the final products result from the regioselective attack of the nucleophiles on the C(1) atom of the three-membered ring of the cation **1a**.

The interest in these compounds, especially with heteroaryl substituents in their molecules, may stem from the peculiarities of their chemical behavior due to the mutual effects of the ferrocene system and the heterocyclic fragment [8]. These effects may result in the emergence of diverse valuable properties, such as biological activity, dyeing ability, possible use as propellant additives or light-sensitive materials, redox switching receptors in supramolecular chemistry, *etc.*, which have previously been observed for a number of heteroarylferrocenes [8].

## 2. Results and Discussion

### 2.1. Synthesis of Diferrocenylmono-, bi-, tricycles and Amino(diferrocenyl)hexa-1,3,5-trienes

**Scheme 2:**
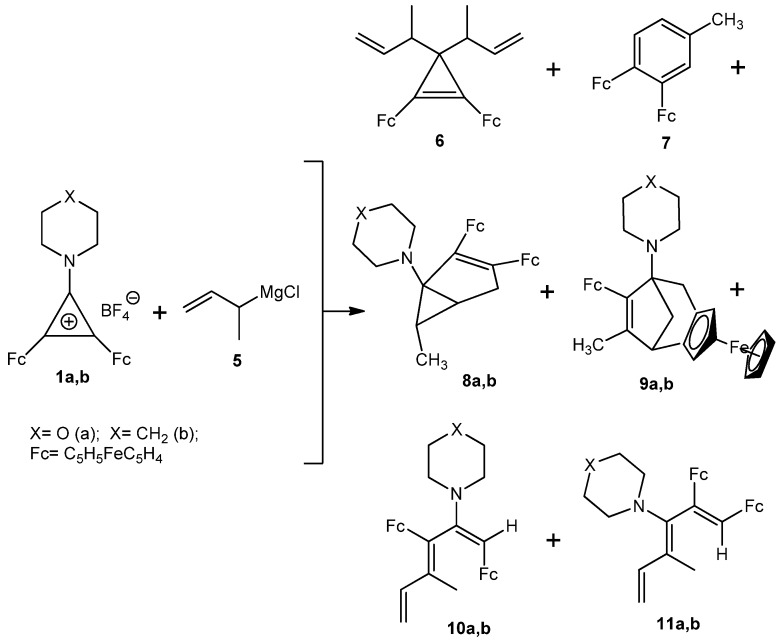
Reaction of 2,3-diferrocenyl-1-morpholino(piperidino)cyclopropenylium tetrafluoroborates **1a**,**b** with 1-methylprop-2-enylmagnesium chloride **5**.

Here we report investigations into the reactions of 2,3-diferrocenyl-1-morpholino(pipe-ridino)cyclopropenylium tetrafluoroborates **1a**, **b** with 1-methylprop-2-enylmagnesium chloride **5** (Scheme 2).

According to a standard procedure [5,6,7], 2,3-diferrocenylcyclopropenone [9] was first converted into 1-ethoxy-2,3-diferrocenylcyclopropenylium tetrafluoroborate and then to tetrafluoroborates **1a**,**b** [1,2], which served as the starting materials in the present study.

We found that tetrafluoroborate **1a** reacted with an excess of 1-methylprop-2-enylmagnesium chloride **5** to afford a complex mixture of reaction products, the main ones being 3,3-bis(1-methylprop-2-enyl)-1,2-diferrocenylcyclopropene (**6**), 3,4-diferrocenyltoluene (**7**), 2,3-diferrocenyl-6-methyl-1-morpholinobicyclo[3.1.0]hex-2-ene (**8a**), 7-ferrocenyl-6-methyl-1-morpholino-3,4-ferrocenobicyclo-[3.2.1]oct-6-ene (**9a**), (*E,E*)*-*1,3-diferrocenyl-4-methyl-2-morpholinohexa-1,3,5-triene (**10a**), and (*Z,E*)*-*1,2-diferrocenyl-4-methyl-3-morpholinohexa-1,3,5-triene (**11a**) (Scheme 2). Analogously tetrafluoroborate **1b** reacted with the Grignard reagent **5** to give compounds **6**, **7**, **8b**, **9b**, **10b**, and **11b** (Scheme 2).

Both reaction mixtures were separated by column chromatography on alumina, and the structures of the isolated products were established based on the data from UV, IR, and ^1^H- and ^13^C-NMR spectroscopy, mass spectrometry, and elemental analysis. The physicochemical characteristics of compounds **6** and **7** corroborate completely their structures.

**Figure 1:**
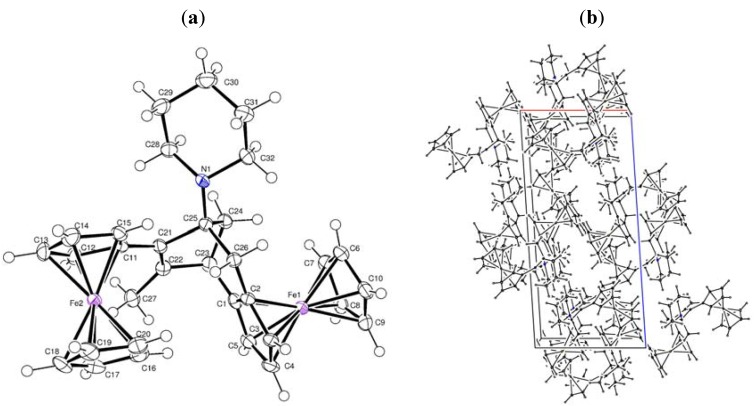
(**a**) X-ray crystal structure of **9b**; (**b**) Crystal packing of **9b**.

We attempted to assign *endo* forms of compounds **8a** and **8b** based on previously reported criteria [10,11,12]. Thus, compounds **8a** and **8b** were characterized as *endo* isomers based on the fact that the signals from three of the protons of the C_5_H_4_ groups are observed at higher field than the singlets from the protons of the C_5_H_5_ groups [10]; the ^1^H-NMR spectra of compounds **8a** and **8b** each contain characteristic doublets for the protons of the methyl group (δ 1.04 and 0.97 ppm), two doublets of doublets for the methylene fragment, two multiplets for the methine fragments (δ 0.94 and 1.77; 0.89 and 1.76 ppm) [11], signals for the protons of the morpholine or piperidine substituents, two singlets for unsubstituted cyclopentadienyl rings and the appropriate number of multiplet signals for two substituted C_5_H_4_ moieties of the ferrocenyl substituents. The ^13^C-NMR spectra of compounds **8a** and **8b** each contained two signals for C_ipso_Fc belonging to two ferrocene fragments, as well as the necessary number of signals for cyclopropyl-fragments δ 13.12, 20.54 ppm (2CH), 20.63 (CH_3_) from **8a**, and δ 12.92, 20.26 (2CH), 21.34 ppm (CH_3_) from **8b** [12]. The position of the heterocyclic substituents at the C(1) carbon atom of the bicycles **8a**,**b** was established based on the data from ^1^H-NMR spectra with a one-dimensional NOE experiment, which showed the interaction of the protons of the two CH groups with the 2-CH_2_-protons of the heterocycles, that completely corroborated their structures. Additional proof of the structures of compounds **9a** and **9b** was obtained from X-ray diffraction analysis of a single crystal of compound **9b**, which was grown by crystallization from CH_2_Cl_2_. A general view of **9b** is shown in Figure 1a, the character of packing of molecules in a crystal is shown in Figure 1b, and the main geometrical parameters are given in Table 1. Data from the X-ray analysis proved the structure of **9b** as 7-ferrocenyl-3,4-ferroceno-6-methyl-1-piperidinobicyclo-[3.2.1]oct-6-ene.

As inferred from the ^1^H-NMR spectra of trienes **10a** and **10b**, they are formed as single isomers. The spectra contained each signals for the olefinic protons of the –CH= fragments and the protons of the terminal =CH_2_ group, singlets for the CH_3_– group and two C_5_H_5_ fragments, as well as multiplets for the protons of the heterocyclic substituents. The data from ^13^C-NMR spectroscopy of these compounds are in full accord with the proposed structures; however, the positions of the ferrocenyl and heterocyclic substituents remained uncertain.

**Table 1:** Selected bond lengths and bond angles for **9b**, **10a** and **10b**.

Selected bond lengths (Å)		Selected bond angles (°)	
	**9b**		
C(21)-C(22)	1.346(4)	C(2)-C(1)-C(23)	117.9(3)
C(22)-C(23)	1.526(4)	C(1)-C(2)-C(26)	122.9(3)
C(23)-C(24)	1.530(4)	C(1)-C(23)-C(22)	105.8(2)
C(24)-C(25)	1.541(4)	C(23)-C(22)-C(21)	109.9(3)
C(25)-C(26)	1.561(4)	C(22)-C(21)-C(25)	109.4(3)
C(25)-N(1)	1.473(4)	C(21)-C(25)-C(26)	106.7(2)
C(26)-C(2)	1.514(4)	C(21)-C(22)-C(27)	130.0(3)
C(1)-C(2)	1.430(4)	C(27)-C(22)-C(23)	119.2(3)
C(22)-C(27)	1.506(4)	C(22)-C(23)-C(24)	101.1(2)
	**10a**		
C(21)-C(22)	1.317(6)	C(21)-C(22)-C(23)	122.8(4)
C(22)-C(23)	1.469(5)	C(22)-C(23)-C(24)	121.8(3)
C(23)-C(24)	1.353(5)	C(23)-C(24)-C(25)	121.3(3)
C(24)-C(25)	1.518(4)	C(24)-C(25)-C(26)	123.2(3)
C(25)-C(26)	1.336(5)	C(22)-C(23)-C(27)	115.8(3)
C(23)-C(27)	1.495(5)	C(24)-C(23)-C(27)	122.4(3)
C(25)-N(1)	1.423(4)	C(24)-C(25)-N(1)	113.6(3)
C(26)-C(1)	1.475(5)	N(1)-C(25)-C(26)	122.7(3)
C(24)-C(11)	1.489(4)	C(25)-C(26)-C(1)	128.7(3)
	**10b**		
C(22)-N(1)	1.414(3)	C(26)-C(25)-C(24)	125.9(2)
C(22)-C(23)	1.501(3)	C(25)-C(24)-C(23)	122.1(2)
C(23)-C(24)	1.343(3)	C(25)-C(24)-C(27)	115.8(2)
C(24)-C(25)	1.468(3)	C(27)-C(24)-C(23)	122.1(2)
C(21)-C(22)	1.343(3)	C(24)-C(23)-C(22)	121.3(2)
C(25)-C(26)	1.326(3)	C(23)-C(22)-C(21)	122.7(2)
C(24)-C(27)	1.510(3)	C(21)-C(22)-N(1)	123.1(2)
C(23)-C(1)	1.480(3)	N(1)-C(22)-C(23)	113.90(19)
C(21)-C(11)	1.462(3)	C(24)-C(23)-C(1)	125.7(2)

**Figure 2:**
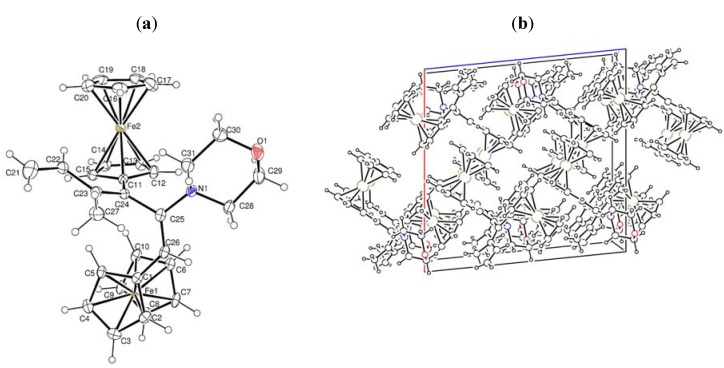
(**a**) X-ray crystal structure of **10a**; (**b**) Crystal packing of **10a**.

**Figure 3:**
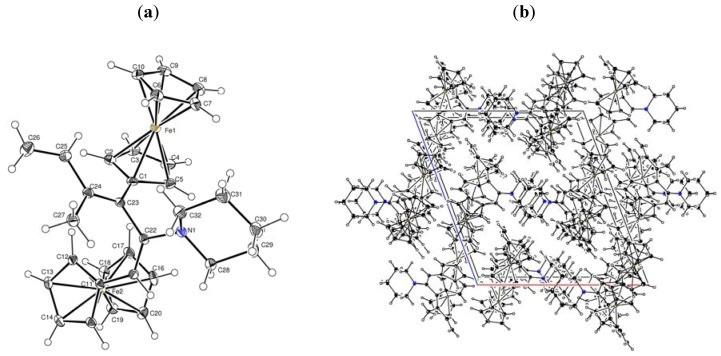
(**a**) X-ray crystal structure of **10b**; (**b**) Crystal packing of **10b**.

To address this issue, we performed X-ray diffraction analysis of their single crystals grown by crystallization from dichloromethane. The general views of the molecules **10a** and **10b** are shown in Figure 2a,b and Figure 3a,b, respectively, and the main geometrical parameters are given in Table 1. Data from the X-ray analysis demonstrated that **10a** is (*E,E*)-1,3-diferrocenyl-4-methyl-2-morpholino-hexa-1,3,5-triene, and **10b** is (*E,E*)-1,3-diferrocenyl-4-methyl-2-piperidinohexa-1,3,5-triene.

Compounds **11a** and **11b** were isolated as single geometrical isomers of (*Z,E*)-1,2-diferrocenyl-3-heteryl-4-methylhexa-1,3,5-trienes as inferred from the following data: the ^1^H and ^13^C-NMR spectra of each of them contain signals for two ferrocenyl, one methyl, and one heterocyclic substituents, two olefinic –CH= and one terminal CH_2_= group. Presumably, the *Z*-arrangement of two ferrocenyl groups at the C(2) = C(1) double bond was assigned based on comparison with *cis-*positions of two Fc-substituents in bicyclic products **8a**,**b**. The *E*-configuration of the C(3) = C(4) double bond was assigned based on comparison with *E*-configuration of the C(3) = C(4) in trienes **10a**,**b**.

**Scheme 3:**
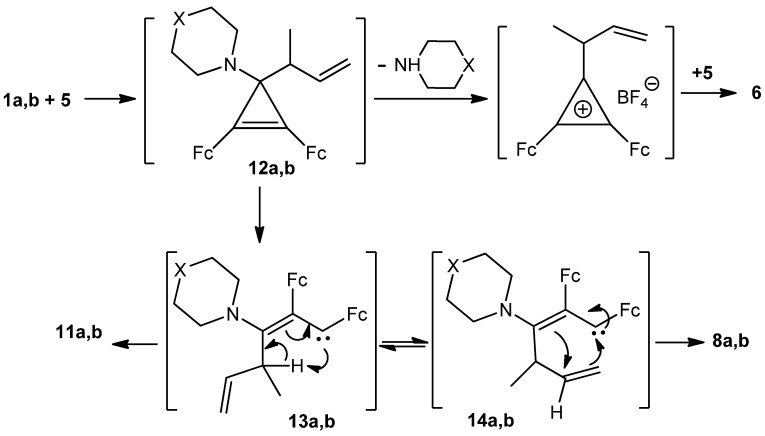
Plausible mechanism of the formation of **6**, **8a**,**b** and **11a**,**b**.

The most feasible mechanism of the formation of compounds **6**, **7**, **8a**,**b**, and **11a**,**b** involves the initial nucleophilic attack of the 1-methylprop-2-enyl anion on the heteroaryl-substituted carbon atom of the three-carbon ring of the cations **1a** and **1b** to afford unstable 1,2-diferrocenyl-3-heteroaryl-3-(1-methylprop-2-enyl)cyclopropenes **12a**,**b** (Scheme 3).

**Scheme 4:**
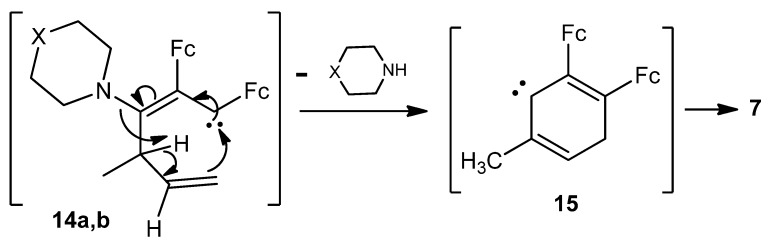
Plausible mechanism of the formation of **7**.

**Scheme 5:**
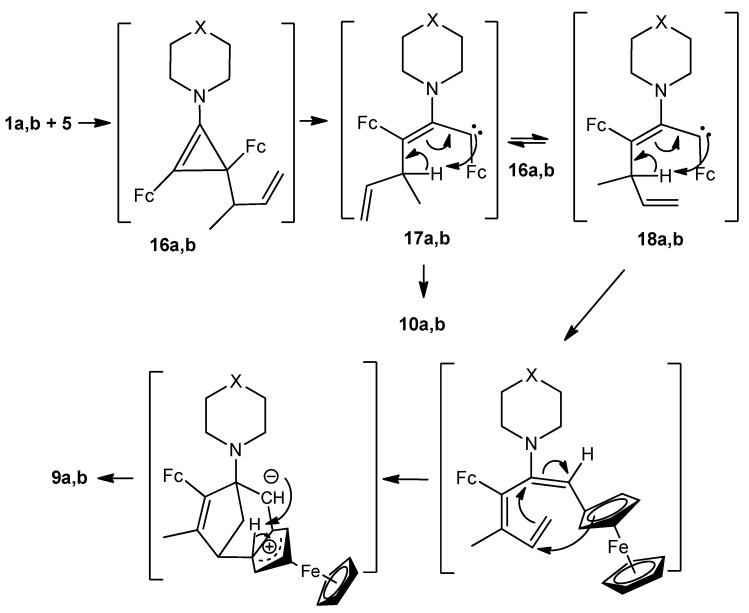
Plausible mechanism of the formation of **9a**,**b** and **10a**,**b**.

The transformation of cyclopropenes with retention of the 3-membered ring and elimination of the heterocyclic substituents results in cyclopropene **6** (Scheme 3). Opening of the cyclopropene ring gives rise to bicyclic **8a**,**b** and linear triene products **11a**,**b** *via* 1,2-diferrocenyl-3-heterylallylcarbenes **13a**,**b**. In our opinion, cyclization of carbenes in their configuration **13a**,**b** leads to bicyclic compounds **8a**,**b**, while it is the intramolecular transformation of these carbenes in the configuration **14a**,**b** results in (*Z,E*)-1,2-diferrocenyl-3-heteryl-4-methylhexa-1,3,5-trienes **11a**,**b** (Scheme 3). On the other hand, intramolecular transformation of carbenes **14a**,**b** with the loss of a heterocyclic substituent (Scheme 4) affords 3,4-diferrocenyltoluene **7** *via* transient 2,3-diferrocenyl-6-methylcyclohexa-2,5-dienylcarbene **15** (Scheme 4). Putative formation mechanisms of compounds **9a**,**b** and **10a**,**b** are depicted in Scheme 5.

The primary nucleophilic attack on the C(2) atom of cyclopropenylium cations **1a**,**b** affords unstable tetrasubstituted cyclopropenes **16a**,**b**. Small-ring opening in these transient species proceeds *via* 1,3-diferrocenyl-2-heterylallylcarbenes **17a**,**b** and **18a**,**b**, their intramolecular transformations result in tricyclic **9a**,**b** and linear **10a**,**b** final products. Compounds **9a**,**b** formed, like bicyclic products **8a**,**b**, from carbenes with configuration **18a**,**b**, while configuration **17a**,**b** of these carbenes gives rise to trienes **10a**,**b**.

### 2.2. Electrochemistry

**Figure 4:**
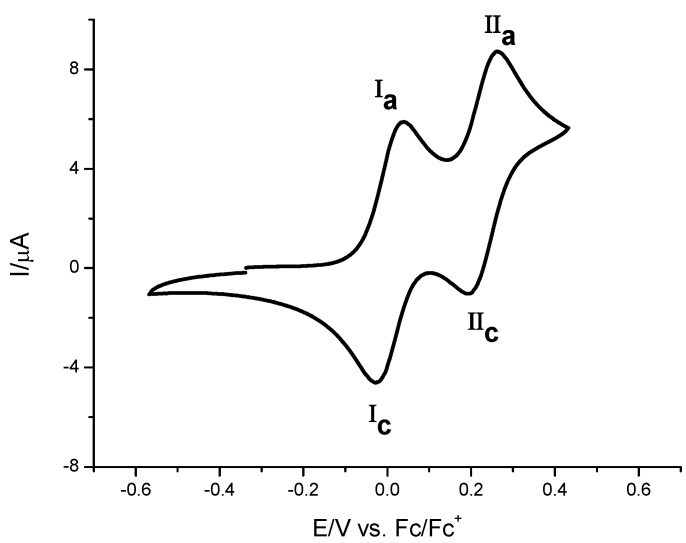
Cyclic voltammograms obtained for **7** in the presence of 0.1 M TBABF_4_ in acetonitrile. Scan rate 0.5 V s^−1^. The scan potential was initiated from E_ocp_ in the positive direction. The working electrode used was a platinum disc.

In the framework of this work, we studied electrochemical behavior of compounds **7**, **8a**, **10a** and **10b** by means of cyclic voltammetry and square wave voltammetry. Figure 4 shows a typical voltammogram of compound **7** obtained in a platinum electrode. Two oxidation signals (**I**_a_ and **II**_a_) with its complementary reduction signals (**I**_c_ and **II**_c_) were detected. The anodic and cathodic peak potential values were independent of scan rate, which indicates the reversibility of the two processes. The logarithmic values of peak current and scan rate were linearly dependent, with a slope near 0.5; this behaviour is characteristic for a diffusion-controlled process. This evidence indicates that processes **I** and **II** are attributed to two consecutive electron transfers of ferrocene moieties.

**Figure 5:**
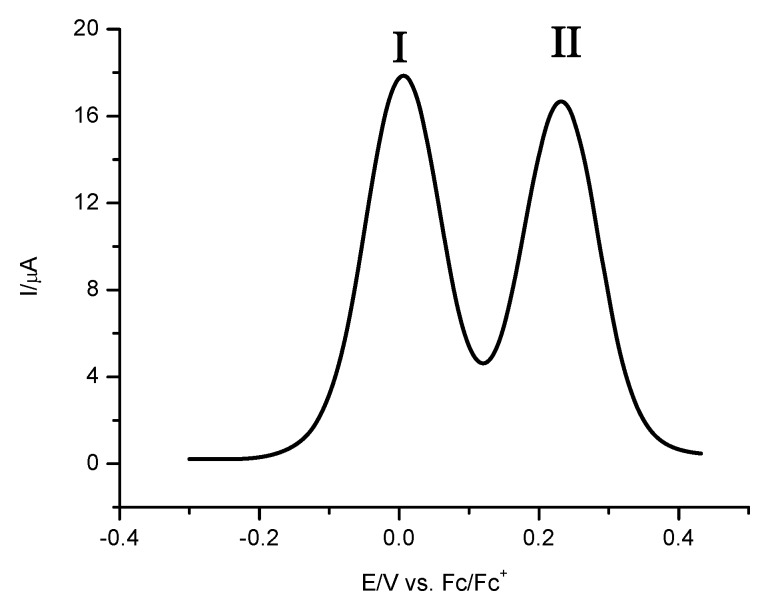
Square wave voltammetry obtained for **7** in the presence of 0.1 M TBABF_4_ in acetonitrile. Amplitude of 50 mV with a frequency of 10 Hz and initiated from E_ocp_ in the positive direction. The working electrode used was a platinum disc.

In order to obtain the formal electrode potential for both processes, square wave voltammetry experiments were carried out (Figure 5). The obtained values were E^0’^ (**I)** = 0.004 V/Fc-Fc^+^ and E^0’^ (**II**) = 0.232 V/Fc-Fc^+^ with its corresponding difference ΔE^0’^ (**II-I**) = 0.228 V and comproportionation constant *K*_com_ of 7151.3 [13,14]. The electrochemical response of **8a** was similar than those described above for **7**.

**Figure 6:**
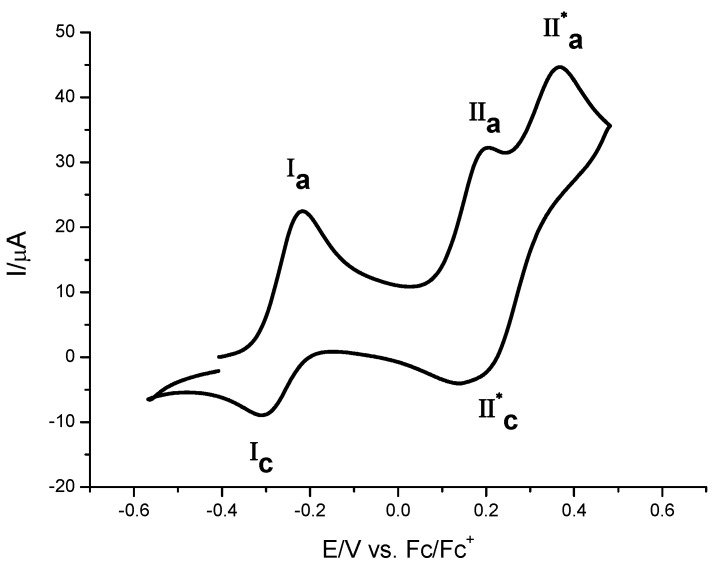
Cyclic voltammograms obtained for **10a** in the presence of 0.1 M TBABF_4_ in acetonitrile. Scan rate 0.5 V s^−1^. The scan potential was initiated from E_ocp_ in the positive direction. The working electrode used was a platinum disc.

The estimated values of E^0’^ (**I)** and E^0’^ (**II**) were 0.058 and = 0.159 V/Fc-Fc^+^ with ΔE^0’^ (**II-I**) = 0.101 V and *K*_com_= 51.4. The highest value of ΔE^0’^ (**II-I**) for **7**, compared with the obtained value for **8a**, can be explained in terms of an increase in the electron density in ferrocene moieties due to the presence of an aromatic ring and the activating properties of the methyl substituent in **7**. This fact suggests that in the *K*_com_ values electron withdrawing an electron donating effects must be consider. Also, the difference in the conjugation manner in compounds **7** and **8a** could be the reason for the difference in ΔE^0’^ (**II-I**) values. Figure 6 presents cyclic voltammoamperometric response of compound **10a**. When the potential scan was initiated in the positive direction, three oxidation signals (**I**_a_, **II**’_a_, and **II**’’_a_) were observed, and when the potential scan was reversed, two reduction signals (**I**_c_ and **II**’_c_) were also observed. We propose that the electrochemical process **I** with its corresponding oxidation and reduction signals, **I**_a_ and **I**_c,_ are attributed to electron transfer ferronece/ferricium^+^ for both moieties. This is in agreement with the fact that a single electrochemical process is observed when there is no electronic communication between two redox centers, due to the long distance within a molecule, as occurs in compound **10a** (Figure 6).

**Figure 7:**
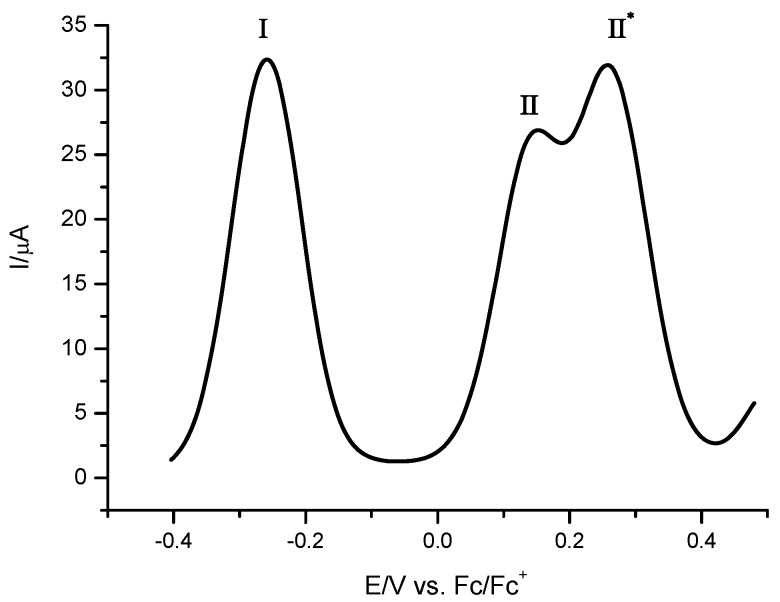
Square wave voltammetry obtained for **10a** in the presence of 0.1 M TBABF_4_ in acetonitrile. Amplitude of 50 mV with a frequency of 10 Hz and initiated from E_ocp_ in the positive direction. The working electrode used was a platinum disc.

On the other hand, based on studies reported previously for molecules with conjugated double bonds*,* the processes **II**’_a_ and **II**’’_a_ can be related to electrochemical generation of the monocation and dication species [15,16,17,18,19,20,21]. Controlled potential coulommetry experiments acquired at potential step corresponding to anodic peak potential values for processes **I**, and **II**, led us to calculate the value of two electrons transferred for each process. The formal electrode potential values were evaluated also using square wave voltammetry experiments (Figure 7).

The values for E^0’^ (**I)**, E^0’^ (**II**), and E^0’^ (II^*^) were −0.260, 0.152, and 0.256 V/Fc-Fc^+^, respectively. The electrochemical response of compound **10b** is similar that obtained for **10a**. However, different values of formal electrode potentials were observed. Table 2 shows a summary of electrochemical behaviour of compounds **7**, **8a**, **10a** and **10b**.

**Table 2:** Formal electrode potential E^0^ (I), E^0^ (II) and Δ E^0^ (II-I), and constant *K*_com_ for compounds **7**, **8a**, **10a**, and **10b**.

**Compound**	E^0^(Ι) ^a^	E^0^ (II) ^a^	E^0^ (ΙΙ^∗^) ^b^	E^0^ (ΙΙ^∗^) ^c^
**7**	0.004	0.232		
**8a**	0.058	0.101V		
**10a **	−0.260 ^d^		0.152	0.256
**10b**	−0.328^ d^		0.136	0.240

## 3. Experimental

### 3.1. General

All the solvents were dried according to standard procedures [22] and were freshly distilled before use. Column chromatography and TLC were carried out on alumina (Brockmann activity III). The ^1^H and ^13^C-NMR spectra of the compounds **6**, **7** **8a**,**b**, **11a**,**b** were recorded on a Unity Inova Varian spectrometer (300 and 75 MHz) for solutions in CDCl_3_, with Me_4_Si as the internal standard. The IR spectra were measured with an FTIR spectrophotometer (Spectrum RXI Perkin-Elmer instruments) using KBr pellets. UV spectra were recorded on a Specord UV-VIS spectrophotometer.The mass spectra were obtained on a Varian MAT CH-6 instrument (EI MS, 70 eV). A LECO CHNS-900 Elementar Analysensysteme was used for elemental analyses.

Electrochemical measurements were carried out in acetonitrile containing 0.1 M tetra-*n-*butyl-ammonium tetrafluoroborate (TBABF_4_) with sample concentration c.a. 1 mM. An Epsilon-BAS potentiostat/galvanostat was used for all experiments. A typical three-electrode array was employed; using a platinum disk as working electrode and a platinum wire as counter-electrode. A silver wire immersed in acetonitrile solution with 0.1 M tetra-*n*-butylammonium chloride (TBACl) was used as a pseudo reference electrode. Before each measurement all solutions were bubbled with nitrogen. Cyclic voltammetry experiments were acquired from the open circuit potential (E_ocp_) to positive direction, at different scan rates (from 0.1 to 0.5 V s^−1^). Square wave voltammetry experiments using amplitude of 50 mV with a frequency of 10 Hz were also performed. All potentials were reported versus the Fc/Fc^+^ couple, according to the IUPAC convention [23].

The following reagents were purchased from Aldrich: Tetrachlorocycloropropene, 98%; ferrocene, 98%; triethyloxonium tetrafluoroborate, 1.0 M solution in dichloromethane; morpholine, 99.5%; piperidine, 99%; 1-methyl-2-propenylmagnesium chloride, 0.5M solution in tetrahydrofuran. *Ethoxy-(diferrocenyl)cyclopropenylium tetrafluoroborate* was obtained by treating 2,3-diferrocenylcyclo-propenone [2] in dichloromethane with triethyloxonium tetrafluoroborate. *Diferrocenyl(morpholino)- or (piperidino)cyclopropenylium tetrafluoroborates* **1a**,**b** were obtained from ethoxy(diferrocenyl)-cyclopropenylium tetrafluoroborate and morpholine or piperidine in dichloromethane [1].

*Reactions of diferrocenyl(morpholino)- or (piperidino)cyclopropenylium tetrafluoroborates* **1a**,**b** *with 1-methyl-2-propenylmagnesium chloride* (**5**). 1-Methyl-2-propenylmagnesium chloride (**5**, 20.0 mL of a 0.5 M solution in tetrahydrofuran) was added to a suspension of salt **1a** or **1b** (5.0 mmol) in dry benzene (100 mL) and the mixture was stirred in an inert dry atmosphere (~70–80 °C) until complete dissolution of the salts **1a**,**b** occurred (~6–8 h). The excess of the organometallic compound was quenched with water (20 mL), the organic layer was separated, concentrated, and the residue was chromatographed on alumina (Brockmann activity III, hexane-dichloromethane, 7:1) to give compounds **6**, **7**, **8a**–**11a** and **8b**–**11b**.

*Reactions of diferrocenyl(morpholino)- or (piperidino)cyclopropenylium tetrafluoroborates* **1a**,**b** *with 1-methyl-2-propenylmagnesium chloride* (**5**). 1-Methyl-2-propenylmagnesium chloride (**5**, 20.0 mL of a 0.5 M solution in tetrahydrofuran) was added to a suspension of salt **1a** or **1b** (5.0 mmol) in dry benzene (100 mL) and the mixture was stirred in an inert dry atmosphere (~70–80 °C) until complete dissolution of the salts **1a**,**b** occurred (~6–8 h). The excess of the organometallic compound was quenched with water (20 mL), the organic layer was separated, concentrated, and the residue was chromatographed on alumina (Brockmann activity III, hexane-dichloromethane, 7:1) to give compounds **6**, **7**, **8a**–**11a** and **8b**–**11b**.

*1,2-Diferrocenyl-3,3-di-(3-buten-2-yl)cyclopropene* (**6**). Orange powder, yield: 0.18–0.26 g (7%–10%), m.p. 103–104 °C (hexane); ^1^H-NMR: 0.88 (d, 3H, CH_3_, *J* = 6.9 Hz), 1.11 (d, 3H, CH_3_, *J* = 6.9 Hz), 2.65 (m, 2H, 2CH), 4.19 (s, 5H, C_5_H_5_), 4.21 (s, 5H, C_5_H_5_), 4.34 (m, 3H, C_5_H_4_), 4.36 (m, 3H, C_5_H_4_), 4.52 (m, 2H, C_5_H_4_), 4.97–5.04 (m, 4H, 2CH_2_=), 5.84–5.98 (m, 2H, 2CH=); ^13^C-NMR: 18.63, 18.81 (2CH_3_), 42.66, 43.19 (2CH), 45.06 (C), 69.32, 69.37 (2C_5_H_5_), 68.91, 69.00, 69.04, 69.08, 69.27, 69.42, 69.44, 69.46 (2C_5_H_4_), 76.04 (2C*_ipso_*Fc), 112.58, 112.88 (2CH_2_=), 144.87, 144.95 (2CH=), 144.56 (2C); MS: *m / z* 516 [M]^+^. Anal. Calcd. for C_31_H_32_Fe_2_: C, 72.11; H, 6.25; Fe, 21.64%. Found: C, 72.23; H, 6.17; Fe, 21.81.

*3,4-Diferrocenyltoluene* (**7**). Orange powder, yield: 0.12–0.18 g (5%–8%), m.p. 124–125 °C; ^1^H-NMR: 2.40 (s, 3H, CH_3_), 4.00 (s, 5H, C_5_H_5_), 4.02 (s, 5H, C_5_H_5_), 4.04 (m, 2H, C_5_H_4_), 4.06 (m, 2H, C_5_H_4_), 4.09 (m, 4H, C_5_H_4_), 7.03 (d, 1H, C_6_H_3_*J* = 8.4 Hz), 7.55 (s, 1H, C_6_H_3_), 7.62 (d, 1H, C_6_H_3_*J* = 8.4 Hz); ^13^C-NMR: 21.17 (CH_3_), 69.32, 69.37 (2C_5_H_5_), 67.08, 6715, 70.56, 70.65 (2C_5_H_4_), 87.81, 87.83 (2C*_ipso_*Fc), 126.54, 131.24, 131.87 (C_6_H_3_), 134.28, 134.93, 136.99 (3C*_ipso_*); MS: *m / z* 460 [M]^+^. Anal. Calcd. for C_27_H_24_Fe_2_: C, 70.47; H, 5.26; Fe, 24.27. Found: C, 70.36; H, 5.40; Fe, 24.15.

*2,3-Diferrocenyl-6-methyl-1-morpholinobicyclo[3.1.0]hex-2-ene* (**8a**). Orange oil, yield: 0.27 g (10%); IR (KBr): 484, 755, 817, 876, 906, 969, 1002, 1031, 1044, 1106, 1119, 1172, 1220, 1263, 1302, 1360, 1449, 1614, 1714, 2250, 2734, 2851, 2955, 3093 cm^−1^; ^1^H-NMR: 0.94 (m, 1H, CH), 1.04 (d, 3H, CH_3_, *J* = 6.9 Hz), 1.77 (m, 1H, CH), 2.35 (dd, 1H, CH_2_, *J* = 4.8, 16.5 Hz), 2.63 (dd, 1H, CH_2_, *J* = 6.6, 16.5 Hz), 2.78 (m, 4H, 2 CH_2_), 3.48 (m, 4H, 2 CH_2_), 3.91 (s, 5H, C_5_H_5_), 4.00 (s, 5H, C_5_H_5_), 3.84 (m, 2H, C_5_H_4_), 3.89 (m, 2H, C_5_H_4_), 3.94 (m, 2H, C_5_H_4_), 4.04 (m, 2H, C_5_H_4_); ^13^C-NMR: 13.12, 20.54 (2CH), 20.63 (CH_3_), 34.23 (CH_2_), 40.38, 50.13 (4CH_2_), 65.09 (C), 68.43, 68.75 (2C_5_H_5_), 67.21, 67.42, 67.61, 67.89, 68.52, 68.91, 69.54, 69.71 (2C_5_H_4_), 82.80, 83.14 (2C*_ipso_*Fc), 133.45, 135.12 (2C); MS: *m / z* 547 [M]^+^. Anal. Calcd. for C_31_H_33_Fe_2_NO: C, 68.03; H, 6.08; Fe, 20.41; N, 2.56. Found: C, 67.96; H, 6.13; Fe, 20.55; N, 2.60.

*7-Ferrocenyl-3,4-ferroceno-6-methyl-1-morpholinobicyclo[3.2.1]oct-6-ene* (**9a**). Orange crystals, yield: 0.82 g (30%), m.p. 167–168 °C; IR (KBr): 497, 802, 809, 820, 998, 1025, 1106, 1153, 1304, 1390, 1442, 1465, 1630, 1715, 1768, 2184, 2793, 2918, 2952, 2976, 3075, 3107 cm^−1^; ^1^H-NMR: 1.75 (dd, 1H, CH_2_, *J* = 5.7, 13.8 Hz), 1.85 (s, 3H, CH_3_), 1.98 (dd, 1H, CH_2_, *J* = 8.4, 13.8 Hz), 2.36 (d, 1H, CH_2_, *J* = 12.6 Hz), 2.62 (d, 1H, CH_2_, *J* = 12.6 Hz), 2.70 (m, 2H, CH_2_), 2.93 (m, 1H, CH), 3.66 (m, 4H, 2CH_2_), 3.88 (m, 4H, 2CH_2_), 3.93 (s, 5H, C_5_H_5_), 4.04 (s, 5H, C_5_H_5_), 3.41 (m, 1H), 3.82 (m, 1H), 4.06 (m, 1H), 4.28 (m, 2H), 4.35 (m, 1H), 5.30 (m, 1H) (C_5_H_4_, C_5_H_3_); ^13^C-NMR: 17.01 (CH_3_), 35.16, 36.66 (2CH_2_), 46.31 (CH), 57.42, 67.71 (4 CH_2_), 68.95 (C), 68.68, 69.36 (2C_5_H_5_), 67.14, 67.29, 67.87, 69.49, 70.22, 70.87, 72.91 (C_5_H_4_, C_5_H_3_), 76.83, 80.06, 93.47 (3C*_ipso_*Fc), 136.22, 139.37 (2C); MS: *m / z* 547 [M]^+^. Anal. Calcd. for C_31_H_33_Fe_2_NO: C, 68.03; H, 6.08; Fe, 20.41; N, 2.56. Found: C, 68.14; H, 5.97; Fe, 20.32; N, 2.41.

*E,E-1,3-Diferrocenyl-4-methyl-2-morpholinohexa-1,3,5-triene* (**10a**). Orange crystals, yield: 0.62 g (22%), m.p. 174–176 °C. λ_max_ (CHCl_3_, 20 °C) 211, 282 nm; IR (KBr): 490, 817, 906, 1000, 1026, 1044, 1106, 1119, 1166, 1220, 1276, 1374, 1444, 1581, 1615, 1819, 2049, 2185, 2730, 2816, 2853, 2947, 3079, 3091 cm^−1^; ^1^H-NMR: 1.77 (s, 3H, CH_3_), 2.92 (m, 4H, 2CH_2_), 3.68 (m, 4H, 2CH_2_), 4.00 (s, 5H, C_5_H_5_), 4.14 (s, 5H, C_5_H_5_), 4.05 (m, 2H, C_5_H_4_), 4.17 (m, 1H, C_5_H_4_), 4.22 (m, 1H, C_5_H_4_), 4.24 (m, 1H, C_5_H_4_), 4.33 (m, 1H, C_5_H_4_), 4.35 (m, 1H, C_5_H_4_), 4.37 (m, 1H, C_5_H_4_), 5.11 (s, 1H, CH=), 5.29 (dd, 1H, CH_2_=, *J* = 1.2, 11.1 Hz), 5.35 (dd, 1H, CH_2_=, *J* = 1.2, 17.4 Hz), 7.89 (dd, 1H, CH=, *J* = 11.1, 17.4 Hz); ^13^C-NMR: 16.60 (CH_3_), 48.17, 67.15 (4CH_2_), 69.01, 69.39 (2C_5_H_5_), 67.18, 67.30, 67.62, 67.66, 67.95 (2C), 70.95, 71.52 (2C_5_H_4_), 84.96, 85.23 (2C*_ipso_*Fc), 113.86 (CH_2_=), 100.24, 137.15 (2CH=), 132.68, 134.15, 147.82 (3C); MS: *m / z* 547 [M]^+^. Anal. Calcd. for C_31_H_33_Fe_2_NO: C, 68.03; H, 6.08; Fe, 20.41; N, 2.56. Found: C, 68.09; H, 6.14; Fe, 20.53; N, 2.38.

*Z,E-1,2-Diferrocenyl-4-methyl-3-morpholinohexa-1,3,5-trienes* (**11a**). Orange oil, yield: 0.33 g (12%). λ_max_ (CHCl_3_, 20 °C) 211, 292 nm; IR (KBr): 493, 815, 822, 865, 896, 1000, 1026, 1106, 1119, 1163, 1226, 1249, 1301, 1386, 1446, 1625, 1651, 2298, 2852, 2956, 3091 cm^−1^; ^1^H-NMR: 1.95 (s, 3H, CH_3_), 3.17 (m, 4H, 2CH_2_), 3.60–3.73 (m, 4H, 2CH_2_), 4.01 (s, 5H, C_5_H_5_), 4.08 (s, 5H, C_5_H_5_), 3.97 (m, 2H, C_5_H_4_), 4.20 (m, 2H, C_5_H_4_), 4.28 (m, 2H, C_5_H_4_), 4.37 (m, 2H, C_5_H_4_), 5.15 (dd, 1H, CH_2_=, *J* = 0.9, 11.4 Hz), 5.36 (dd, 1H, CH_2_=, *J* = 0.9, 16.8 Hz), 6.25 (s, 1H, CH=), 7.03 (dd, 1H, CH=, *J* = 11.4, 16.8 Hz); ^13^C-NMR: 15.87 (CH_3_), 45,84, 65.13 (4CH_2_), 68.88, 69.12 (2C_5_H_5_), 68.46, 68.81, 69.34, 70.12 (2C_5_H_4_), 80.76, 82.60 (2C*_ipso_*Fc), 116.72 (CH_2_=), 128.62, 134.06 (2CH=), 132.42, 133.74, 148.13 (3C); MS: *m / z* 547 [M]^+^. Anal. Calcd. for C_31_H_33_Fe_2_NO: C, 68.03; H, 6.08; Fe, 20.41; N, 2.56. Found: C, 67.87; H, 6.01; Fe, 20.31; N, 2.24.

*2,3-Diferrocenyl-6-methyl-1-piperidinobicyclo[3.1.0]hex-2-ene* (**8b**). Orange oil, yield: 0.22 g (8%); IR (KBr): 487, 761, 815, 874, 906, 966, 1001, 1017, 1024, 1103, 1117, 1175, 1214, 1263, 1303, 1360, 1444, 1617, 1716, 2247, 2723, 2867, 2909, 3093 cm^−1^; ^1^H-NMR: 0.89 (m, 1H, CH), 0.97 (d, 3H, CH_3_, *J* = 6.9 Hz), 1.49 (m, 2H, CH_2_), 1.76 (m, 1H, CH), 2.27 (dd, 1H, CH_2_, *J* = 4.5, 16.2 Hz), 2.46 (dd, 1H, CH_2_, *J* = 6.6, 16.2 Hz), 2.63 (m, 4H, 2CH_2_), 3.04 (m, 4H, 2CH_2_), 3.87 (s, 5H, C_5_H_5_), 3.98 (s, 5H, C_5_H_5_), 3.82 (m, 2H, C_5_H_4_), 3.92 (m, 2H, C_5_H_4_), 4.00 (m, 2H, C_5_H_4_), 4.02 (m, 2H, C_5_H_4_); ^13^C-NMR: 12.92, 20.46 (2CH), 21.34 (CH_3_), 32.45 (CH_2_), 47.56 (5CH_2_), 33.87, 40.19 (5CH_2_), 64.17 (C), 68.36, 68.59 (2C_5_H_5_), 67.19, 67.40, 67.54, 67.82, 68.73, 68.99, 69.32, 69.34 (2C_5_H_4_), 82.54, 82.87 (2C*_ipso_*Fc), 132.62, 133.91 (2C); MS: *m / z* 545 [M]^+^. Anal. Calcd. for C_32_H_35_Fe_2_N: C, 70.48; H, 6.47; Fe, 20.48; N, 2.57. Found: C, 70.32; H, 6.54; Fe, 20.31; N, 2.35.

*7-Ferrocenyl-3,4-ferroceno-6-methyl-1-piperidinobicyclo[3.2.1]oct-6-ene* (**9b**). Orange crystals, yield: 0.84 g (31%), m.p. 198–199 °C; IR (KBr): 498, 801, 810, 819, 999, 1024, 1105, 1153, 1307, 1394, 1439, 1464, 1629, 1715, 1764, 2184, 2797, 2912, 2941, 2971, 3075, 3103 cm^−1^; ^1^H-NMR: 0.87 (m, 2H, CH_2_), 1.44 (m, 4H, 2CH_2_), 1.85 (s, 3H, CH_3_), 2.11 (m, 4H, 2CH_2_), 2.21 (dd, 1H, CH_2_, *J* = 5.1, 9.6 Hz), 2.33 (d, 1H, CH_2_, *J* = 9.6 Hz), 2.69 (d, 1H, CH_2_, *J* = 15.6 Hz), 2.92(d, 1H, CH, *J* = 5.1 Hz), 3.27 (d, 1H, CH_2_, *J* = 15.6 Hz), 3.74 (s, 5H, C_5_H_5_), 4.08 (s, 5H, C_5_H_5_), 3.94 (m, 1H), 3.97 (m, 2H), 4.07 (m, 2H), 4.31 (m, 1H), 4.97 (m, 1H), 5.30 (C_5_H_4_, C_5_H_3_); ^13^C-NMR: 15.99 (CH_3_), 25.65, 29.96, 36.54 (3CH_2_), 45.00 (CH), 26.99, 48.44 (4CH_2_), 68.82 (C), 68.68, 69.36 (2C_5_H_5_), 63.14, 64.61, 66.58, 67.12, 67.37, 67.78, 75.07 (C_5_H_4_, C_5_H_3_), 80.34, 83.90, 91.81 (3C*_ipso_*Fc), 131.42, 141.58 (2C); MS: *m / z* 545 [M]^+^. Anal. Calcd. for C_32_H_35_Fe_2_N: C, 70.48; H, 6.47; Fe, 20.48; N, 2.57. Found: C, 70.57; H, 6.32; Fe, 20.53; N, 2.65.

*E,E-1,3-Diferrocenyl-4-methyl-2-piperidinohexa-1,3,5-triene*** (10b**). Orange crystals, yield: 0.57 g (21%), m.p. 206–207 °C; λ_max_ (CHCl_3_, 20 °C) 211, 293 nm; IR (KBr): 492, 819, 903, 1004, 1024, 1046, 1106, 1121, 1163, 1225, 1277, 1375, 1444, 1580, 1621, 1820, 2037, 2191, 2728, 2815, 2839, 2952, 3081, 3093 cm^−1^; ^1^H-NMR: 1.53 (m, 6H, 3CH_2_), 1.74 (s, 3H, CH_3_), 2.96 (m, 4H, 2CH_2_), 3.97 (s, 5H, C_5_H_5_), 4.15 (s, 5H, C_5_H_5_), 4.01 (m, 2H, C_5_H_4_), 4.12 (m, 1H, C_5_H_4_), 4.20 (m, 1H, C_5_H_4_), 4.22 (m, 1H, C_5_H_4_), 4.34 (m, 1H, C_5_H_4_), 4.36 (m, 1H, C_5_H_4_), 4.37 (m, 1H, C_5_H_4_), 5.05 (s, 1H, CH=), 5.24 (dd, 1H, CH_2_=, *J* = 1.5, 11.1 Hz), 5.31 (dd, 1H, CH_2_=, *J* = 1.5, 17.4 Hz), 7.84 (dd, 1H, CH=, *J* = 11.1, 17.4 Hz); ^13^C-NMR: 16.48 (CH_3_), 24.76 (CH_2_), 26.35, 44.86 (4CH_2_), 68.98, 69.39 (2C_5_H_5_), 67.03, 67.43, 67.53, 67.69, 67.90, 69.37, 70.88, 71.78 (2C_5_H_4_), 85.43, 85.91 (2C*_ipso_*Fc), 113.35 (CH_2_=), 99.29, 137.44 (2CH=), 133.50, 133.79, 148.87 (3C); MS: *m / z* 545 [M]^+^. Anal. Calcd. for C_32_H_35_Fe_2_N: C, 70.48; H, 6.47; Fe, 20.48; N, 2.57. Found: C, 70.37; H, 6.58; Fe, 20.61; N, 2.49.

*Z,E-1,2-Diferrocenyl-4-methyl-3-piperidinohexa-1,3,5-trienes* (**11b**). Orange oil, yield: 0.43 g (16%). λ_max_(CHCl_3_, 20 °C) 211, 294 nm; IR (KBr): 489, 812, 818, 860, 896, 1006, 1021, 1103, 1121, 1163, 1224, 1251, 1302, 1385, 1443, 1625, 1648, 2294, 2855, 2962, 3093 cm^−1^; ^1^H-NMR: 1.02–1.21 (m, 2H, CH_2_), 1.34–1.45 (m, 4H, 2CH_2_), 1.90 (s, 3H, CH_3_), 2.35 (m, 4H, 2 CH_2_), 4.00 (s, 5H, C_5_H_5_), 4.09 (s, 5H, C_5_H_5_), 3.85 (m, 2H, C_5_H_4_), 3.93 (m, 2H, C_5_H_4_), 4.13 (m, 2H, C_5_H_4_), 4.22 (m, 2H, C_5_H_4_), 5.12 (dd, 1H, CH_2_=, *J* = 1.2, 11.1 Hz), 5.24 (dd, 1H, CH_2_=, *J* = 1.2, 17.4 Hz), 6.18 (s, 1H, CH=), 6.97 (dd, 1H, CH=, *J* = 11.1, 17.4 Hz); ^13^C-NMR: 15.63, (CH_3_), 20.67 (CH_2_), 25.95, 37.86 (4CH_2_), 68.47, 69.02 (2C_5_H_5_), 68.17, 68.23, 69.25, 70.01 (2C_5_H_4_), 81.04, 82.47 (2C*_ipso_*Fc), 116.74 (CH_2_=), 126.63, 130.13 (2CH=), 131.65, 134.01, 147.29 (3C); MS: *m / z* 545 [M]^+^. Anal. Calcd. for C_32_H_35_Fe_2_N: C, 70.48; H, 6.47; Fe, 20.48; N, 2.57. Found: C, 70.55; H, 6.34; Fe, 20.60; N, 2.61.

### 3.2. Crystal structures of ***9b**, **10a*** and ***10b***

Crystals of **9b**, **10a** and **10b** were obtained from dichloromethane. The unit cell parameters and the X-ray diffraction intensities of **10a** were recorded on a Siemens P4 diffractometer, of **9b** and **10b** on a Gemini (detector Atlas CCD, Cryojet N_2_) diffractometer. The structure of compounds **9b**, **10a** and **10b** were solved by the direct method (SHELXS [24]) and refined using full-matrix least-squares on *F*^2^.

*Crystal data for C_32_H_35_Fe_2_N* (**9b**): M = 545.31 g mol^−1^, monoclinic P21/n, *a* = 10.5528(9), b = 10.3965(9), c = 22.888(2) Å, α = 90, β = 93.605(10), γ = 90^o^, V = 2506.1(4) Å^3^, T = 130(2) K, Z = 4,ρ = 1.445 Mg/m^3^, λ (Mo − Kα) = 0.71073 Å, F(000) = 1144, absorption coefficient 9.429 mm^−1^, crystal size 0.444 × 0.2658 × 0.1397 mm^3^, index ranges −12 ≤ h ≤ 12, −12 ≤ k ≤ 8, −27 ≤ l ≤ 27, scan range 3.87 ≤ *θ* ≤ 68.09^o^, 4574 independent reflections, R_int_ = 0.0757, 14354 total reflections, 318 refinable parameters, final R indices [I > 2σ(I)] R_1_ = 0.0487, wR_2_ = 0.1299, R indices (all data) R_1_ = 0.0588, wR_2_ = 0.1388, goodness-of-fit on F^2^ 1.083, largest difference peak and hole 0.482/−0.915 eÅ^−3^.

*Crystal data for* *C_31_H_33_Fe_2_NO* (**10a**): M = 547.28 g mol^−1^, monoclinic P21/c, *a* = 15.602(1), b = 10.598(2), c = 15.362(1) Å, α = 90, β = 95.844(5), γ = 90^o^, V = 2526.9(5) Å^3^, T = 298(2) K, Z = 4, ρ = 1.439 Mg/m^3^, λ (Mo − Kα) = 0.71073 Å, F(000) = 1144, absorption coefficient 1.173 mm^−1^, crystal size 0.6003 × 0.4151 × 0.0545 mm^3^, index ranges −15 ≤ h ≤ 19, −13 ≤ k ≤ 9, −19 ≤ l ≤ 18, scan range 3.25 ≤ *θ* ≤ 26.62^o^, 5270 independent reflections, R_int_ = 0.0456, 11939 total reflections, 317 refinable parameters, final R indices [I > 2σ(I)] R_1_ = 0.0551, wR_2_ = 0.1644, R indices (all data) R_1_ = 0.0715, wR_2_ = 0.1781, goodness-of-fit on F^2^ 1.090, largest difference peak and hole 1.348/−0.600 eÅ^−3^.

*Crystal data for* *C_32_H_35_Fe_2_N* (**10b**): M = 545.31 g mol^−1^, monoclinic P21/c, *a* = 15.5360(5), b = 10.2960(2), c = 16.8430(5) Å, α = 90, β = 110.613(3), γ = 90^o^, V = 2521.70(12) Å^3^, T = 130(2) K, Z = 4, ρ = 1.436 Mg/m^3^, λ (Mo − Kα) = 0.71073 Å, F(000) = 1144, absorption coefficient 1.172 mm^−1^, crystal size 0.5175 × 0.1764 × 0.1143 mm^3^, index ranges −15 ≤ h ≤ 19, −11 ≤ k ≤ 12, −20 ≤ l ≤ 20, scan range 3.43 ≤ *θ* ≤ 26.06^o^, 4978 independent reflections, R_int_ = 0.0442, 18609 total reflections, 317 refinable parameters, final R indices [I > 2σ(I)] R_1_ = 0.0382, wR_2_ = 0.0979, R indices (all data) R_1_ = 0.0502, wR_2_ = 0.1020, goodness-of-fit on F^2^ 0.990, largest difference peak and hole 0.830/−0.498 eÅ^−3^.

CCDC-804034 (for **9b**), CCDC-804035 (for **10a**) and CCDC-804036 (for **10b**) contain the supplementary crystallographic data for this paper. These data can be obtained free of charge at www.ccdc.cam.ac.uk/const/retrieving.html [or from the Cambridge Crystallographic Data Centre, 12, Union Road, Cambrige CB2 IEZ, UK (Fax: +44-1223-336033; Email: deposit@ccdc.cam.ac.uk or www: http://www.ccdc.cam.ac.uk).

## 4. Conclusions

The reactions of 1-amino-2,3-diferrocenylcyclopropenylium cations **1a**,**b** with strong C-nucleophiles are nonregioselective (unlike the reactions with β-dicarbonyl compounds). The carbanions attack the C(1) and C(2) atoms of the cyclopropenylium cations with equal probability to afford two types of tetrasubstituted cyclopropenes, *viz*., 3-amino-1,2-diferrocenyl- and 1-amino-2,3-diferrocenylcyclopropenes. Intramolecular transformations of the latter formed due to opening of the three-membered ring giving rise to two types of diferrocenylvinylcarbene species **13a**,**b**, **14a**,**b**, **15** and **17a**,**b**, **18a**,**b** differing in location of ferrocenyl, methyl, allyl and heteroaryl substituents in the carbon chain. Their intramolecular conversions to cyclic, bicyclic, tricyclic and linear final products certainly form the basis for further investigations in the fields of theoretical and synthetic organic chemistry, the search for new methods for an access to practically valuable materials.

The electrochemical behaviour of compounds **7**, **8a**, **10a** and **10b** was investigated by means of cyclic voltammetry and square wave voltammetry. For **7** and **8a** two electrochemical processes (I-II), attributed to the oxidation of the ferrocene moieties were found. On the other hand for compounds **10a** and **10b** a single electron transfer for both ferrocene groups and the electrochemical generation of the monocation and dication species were detected.
